# Sulfur's Long Game: 145 Years of Soil Sulfur Speciation in the World's Oldest Agricultural Experiments

**DOI:** 10.1111/gcb.70136

**Published:** 2025-03-20

**Authors:** Meghan Barnard, Brigid A. McKenna, Ram C. Dalal, Steve P. McGrath, Zhe H. Weng, Jeremy L. Wykes, Peter M. Kopittke

**Affiliations:** ^1^ School of Agriculture and Food Sustainability The University of Queensland St Lucia Queensland Australia; ^2^ Sustainable Soils and Crops Rothamsted Research Harpenden UK; ^3^ School of Agriculture, Food, and Wine The University of Adelaide Urrbrae South Australia Australia; ^4^ The Australian Synchrotron Clayton Victoria Australia

**Keywords:** Broadbalk winter wheat experiment, farmyard manure, land management, organic amendment, soil sulfur, wilderness regeneration

## Abstract

Sulfur (S) is an essential plant nutrient, but ongoing decreases in inorganic S inputs to soil continue to reduce S availability in agricultural soils globally. This study investigated long‐term trends in soil S speciation after land use change and the application of different soil amendments using the world's longest‐running agricultural experiments at the Rothamsted Research Centre, UK. Soil samples spanning 145 years were obtained from the Broadbalk Wheat Experiment (continuous cropping with different amendments) and two Wilderness sites, Broadbalk Wilderness and Geescroft Wilderness (cropping land left to rewild) and analysed using synchrotron‐based x‐ray absorption near‐edge structure (XANES) spectroscopy. It was found that changes in S speciation were linked to changes in soil organic carbon (SOC). In the Broadbalk Winter Wheat experiment, farmyard manure applications increased the proportion of reduced C‐bonded S by 40% over 145 years, while the S speciation in the inorganic fertiliser (NPKMgS) and Control treatments remained unchanged and was comprised of ~48% oxidised S. In the Wilderness sites (cropping ceased 143–146 years from present), SOC accumulation during rewilding generally increased the proportions of reduced organic S. However, soil acidification at the Geescroft site initially increased the average oxidation state of S (from +3.7 in 1883 to +4.4 in 1965) despite increasing SOC. Thus, whilst SOC is important in controlling S speciation, soil pH also has a central effect. These findings provide new insights into the long‐term dynamics of S speciation under different agricultural practices and land‐use changes and contribute to our understanding of S and its availability in cropping systems.

## Graphical abstract

This study investigated long‐term trends in soil sulfur (S) speciation after land use change and application of different soil amendments using the Broadbalk Winter Wheat Experiments and Broadbalk and Geescroft Wilderness sites at the Rothamsted Research Centre, UK. Changes in S speciation were linked to land management practices that altered total soil organic carbon content (SOC), highlighting the interconnectivity of SOC and S cycling in soils. This study provides new insights into the long‐term dynamics of S speciation under different agricultural practices and land‐use changes, contributing to our understanding of S and its availability in cropping systems.

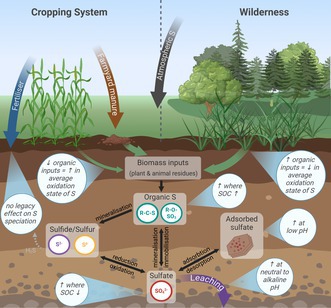

## 1 Introduction

Sulfur (S) is one of the four essential macronutrients for plant growth and is necessary for many functions such as the synthesis of chlorophyll and the formation of essential amino acids such as cysteine and methionine (Narayan et al. 2023). Within the soil environment, S occurs in both inorganic and organic forms, with plants taking up S in the inorganic form as sulfate (Scherer 2009). Although 95%–98% of S is in the organic form in most soil environments (Solomon et al. 2011), S can be converted from organic to plant‐available (inorganic) form through mineralisation and oxidising reactions facilitated by various soil microbes. Organic S is thus important for the supply of S for plant growth, especially since inorganic S inputs are declining in agricultural systems due to the increased regulation of atmospheric emissions, the increased use of low S‐containing fertilisers, and the ongoing removal of S in agricultural products (Eriksen 2009). Accordingly, cases of S deficiency in grassland and arable crops have been reported throughout the last few decades (Dobrokhotov et al. 2023; Solberg et al. 2007; Yu et al. 2021; Zhao and McGrath 1994), with the incidence of S deficiency increasing substantially since the 2000s and expected to worsen (Sharma et al. 2024). This threatens both crop yield and crop quality, for example, since S plays a key role in the protein content of wheat and the production of oil in oilseed crops (Prasad and Shivay 2016; Zenda et al. 2021). As a result, the demand and use of S fertilisers have been steadily increasing globally, including within the United Kingdom (Department for Environment, F. R. A 2022; Bothare 2024; Gerson and Hinckley 2023). The need for appropriate management of S in agriculture is thus vital, especially to avoid reliance on fertiliser, which can be damaging to the receiving environment, as has been learnt from nitrogen (N) and phosphorus (P) (Feinberg et al. 2021; Hinckley and Driscoll 2022). Indeed, Gerson and Hinckley (2023) discuss the need to further develop our understanding of the biogeochemical cycling of S to inform management strategies to reduce reliance on mineral fertilisers.

Soil S is generally grouped into two broad forms—organic S and inorganic S—with transformations controlled by a range of biotic and abiotic factors (Lucheta and Lambais 2012). Moreover, the chemistry of S within the soil environment is complex, with S oxidation states ranging from −2 to +6, enabling it to form a wide range of compounds with different chemical functions and properties (Solomon et al. 2011). Organic S exists as either C‐bonded S (i.e., S directly bonded to C) or as sulfate ester‐S (i.e., S indirectly bonded to C) (Scherer 2009). This C‐bonded S can be transformed to inorganic sulfate via in vivo biological mineralisation and is governed by the microbial demand for energy. In contrast, sulfate ester‐S is transformed via ex vivo biochemical mineralisation during hydrolysis by extracellular sulfatase enzymes and is governed by the microbial demand for S (McGill and Cole 1981). These two broad fractions of organic S (C‐bonded S and sulfate ester‐S) were traditionally isolated based on their reactivity with hydroiodic acid (Eriksen 2009). However, this technique has limitations, as the complexity of organic S may not be adequately identified by only separating two broad groups of organic S (Kertesz and Mirleau 2004; Solomon et al. 2003; Zhao et al. 2006).

Given that the majority of S in soil exists in organic form, S availability is thus intrinsically linked to soil organic matter (SOM) and the cycling of soil organic carbon (SOC) (Chapman 1997). Indeed, many studies have demonstrated a positive correlation between SOC and S (Ghosh et al. 2022; Kopittke et al. 2016; Kumar et al. 2022). Total SOC stocks are known to be sensitive to land use change and different land management strategies (Guo and Gifford 2002; Murty et al. 2002; Poeplau and Don 2013). Changes in SOC stocks can thus subsequently influence the S supply in soils. However, there is still a need to improve our understanding of how these changes to SOC translate to changes in the S chemistry of soil, especially over long‐time scales (> 100 years). Addressing this knowledge gap will enable improved management of the organic forms of S, which can serve as a source of plant‐available S. One opportunity in this regard is to examine the classical agricultural experiments at the Rothamsted Research Centre (UK) which has the world's longest agronomic experiments. Although present‐day investigations are confined by the original design of these agricultural experiments, the long‐term nature of the experiment allows researchers to gain valuable insights that are not possible elsewhere. For example, these trials have enabled researchers to develop an invaluable body of knowledge about many aspects of soil science (Fornara et al. 2011; Ma et al. 2020; Poulton et al. 2024) and more specifically, soil S dynamics (Knights et al. 2001, 2000; Zhao et al. 2006).

Owing to the complexity of soil S, synchrotron‐based x‐ray absorption near‐edge structure (XANES) spectroscopy has been identified as an effective method of investigating S‐containing organic functional groups. The advantage of XANES spectroscopy is that it is sensitive to the bonding environment of S and can thus identify specific functional groups of S across its full range of oxidation states. Additionally, XANES can be undertaken directly in situ, preventing the risk of introducing experimental artefacts that may occur when using operationally defined chemical separation techniques (Manceau and Nagy 2012; Prietzel et al. 2007; Solomon et al. 2003). Despite this, however, most studies that have used XANES to examine S speciation in soils have used humic extracts rather than in situ analyses of the soil (humic extracts are more concentrated and are thus easier to analyse), but such extraction procedures potentially result in experimental artefacts by modifying S speciation to a point that it is no longer representative of the bulk soil (Eriksen 2009; Prietzel et al. 2007; Zhao et al. 2006).

In the current study, we aimed to examine the effects of long‐term land‐use change on S speciation. For this, we used The Broadbalk Classical Wheat Experiment and the Geescroft and Broadbalk Wilderness Regeneration trials to explore long‐term S dynamics in cropping systems with different nutrient treatments as well as in rewilding ecosystems where cropped land is reverted to wilderness. The benefit of using this long‐term experiment is that it identifies clear trends in S pools rather than the short‐term fluctuations in soil S (Knights et al. 2000). In addition to conventional approaches, we utilized synchrotron‐based XANES analyses to examine S speciation in situ. We hypothesized that within the Broadbalk Classical Wheat Experiment, the different nutrient treatments would exert more influence on S speciation than the changes in SOC caused by cropping. Specifically, S would become more oxidized in all treatments over time due to ongoing disturbance (Solomon et al. 2005), but where farmyard manure (FYM) was applied, the increase in oxidized S would be smaller than with mineral fertilizer additions of N, P, potassium (K), magnesium (Mg), and S (referred to as NPKMgS) as manure amendments have been shown to increase proportions of reduced C‐bonded S (Castellano and Dick 1988). Within the two Wilderness Regeneration Sites where cropped land was converted into regenerated wilderness areas, it was hypothesized that the trends in S speciation would be opposite to what occurs in the long‐term wheat experiment, with the proportion of reduced organic S increasing as SOC stocks are replenished. This is because increasing SOC stocks have been linked to greater proportions of reduced S in previous studies (Xu et al. 2016).

## 2 Materials and Methods

### 2.1 Sampling Location

Bulk soil samples were collected from two experimental systems from 0 to 23 cm depth and archived at Rothamsted Research, UK. The strategy for sample selection aimed to optimize information gained from these long‐term trials and capture the long‐term temporal variations while minimizing the consumption of the archived sample set. The first system was the Broadbalk Winter Wheat Experiment in continuous winter wheat ( *Triticum aestivum* ) (51°48′34.1″N 0°22′22.8″W) with samples collected in 1865, 1914, 1944, 1987, and 2010 (i.e., over 145 years). Soil found within the Broadbalk Winter Wheat Experiment is classed as a Chromic Luvisol, comprising 19%–39% clay with clay‐with‐flints (Rothamsted Research 2024). We examined three treatments at each of these time points: (1) unamended (Control, Plot 3), (2) FYM additions (Plot 2.2) which receive 35 t ha^−1^ fresh weight of FYM annually, and (3) NPKMgS fertilizer additions (Plot 8) which receive 144 kg N ha^−1^, 35 kg P ha^−1^ (although P additions were paused in 2000 due to high levels of soil P), 90 kg K ha,^−1^ and 12 kg Mg ha^−1^ annually. The amount of S applied via NPKMgS fertilizer has varied over time but overall has declined as purer N fertilizers were used. Atmospheric S deposition would have also contributed to soil S, peaking annually at ca. 65 kg S ha^−1^ in the 1970s and decreasing to ca. 5 kg S ha^−1^ by the 2000s (McGrath et al. 2002). Between 2000 and 2010, S was applied at 0 kg S ha^−1^ to the Control, 40 kg S ha^−1^ to the FYM treatment (sourced from the FYM), and 53 kg S ha^−1^ to the NPKMgS treatment. The average annual S outputs through grain removal between 2000 and 2010 have been estimated as 1 kg S ha^−1^ for the Control, 7 kg S ha^−1^ for the NPKMgS, and 6 kg S ha^−1^ for the FYM (Glendining et al. 2024; McGrath et al. 2002; Perryman et al. 2023; Rothamsted Research 2021b). Although the experiment started from 1843 onwards, it should be noted that arable cropping at the site of the Broadbalk Winter Wheat Experiment had already occurred for centuries before the start of the experiment (Macdonald et al. 2018).

In addition to the Broadbalk Winter Wheat Experiment, we also investigated bulk soil samples from two regenerated wilderness areas: (1) Broadbalk Wilderness (samples from 1881, 1904, 1964 and 1999) which is located adjacent to the Broadbalk Winter Wheat Experiment (51°48′35.4″N 0°22′31.1″W), and (2) Geescroft Wilderness (samples from 1883, 1904, 1965, and 1999), which is located approximately 1.2 km from the Broadbalk site (51°48′07.9″N 0°21′37.3″W). Soil at the Broadbalk Wilderness and Geescroft Wilderness sites is also Chromic Luvisols (Rothamsted Research 2024b, 2024c). Before woodland regeneration started in 1882, the Broadbalk Wilderness site was used for continuous winter wheat between 1843 and 1881 and had been heavily limed before 1843, while woodland regeneration at the Geescroft site started in 1878 after being used for long‐term cultivation of faba bean ( *Vicia faba* ) between 1847 and 1878 and was not limed. Vegetation at the Broadbalk Wilderness site is now mostly comprised of ash ( *Fraxinus excelsior* ), sycamore ( *Acer pseudoplatanus* ), and hawthorn (*Craetagus monogyna*). The Geescroft site is now a deciduous woodland dominated by oak ( *Quercus robur* ) (Poulton et al. 2003).

### 2.2 Total SOC, N, and S

Total organic C and total N values were sourced from the electronic Rothamsted Archive (Perryman 2015a, 2015b; Rothamsted Research 2018, 2021a). Total S was determined following an aqua regia (80:20 V/V) digestion by inductively‐coupled plasma optical emission spectrometry (ICP‐OES). For extractable S, an extraction was undertaken using 0.016 M KH_2_PO_4_ (Zhao and McGrath 1994) with total extractable S (sulfate‐S and soluble organic S) then determined by ICP‐OES, while ion chromatography (IC) was used for the determination of extractable inorganic sulfate‐S. The concentration of extractable organic S was then determined as the difference between total extractable S and inorganic S.

### 2.3 Synchrotron‐Based XANES

The S speciation in those soil samples was assessed using XANES spectroscopy at the MEX‐2 beamline at the Australian Synchrotron (ANSTO) in Victoria, Australia. The incident X‐ray energy was selected using a Si(111) double crystal monochromator, with a photon flux of 1 × 10^11^ photons s^−1^. The S K‐edge spectra were acquired with a four‐element Si drift detector (Vortex ME4) operating in fluorescence mode under a vacuum of 2.5 × 10^−6^ mbar. The beam was calibrated with Na_2_S_2_O_3_ at 2472.02 eV and was set to a size of 5 mm × 3 mm. The dwell time was 2 s per energy across the entire energy range of 2420 and 2600 eV. Step sizes were 5 eV from 2420 to 2460 eV, 0.5 eV from 2460 to 2466 eV, 0.05 eV from 2466 to 2486 eV, 20 eV from 2486 to 2520 eV, and finally 50 eV from 2520 to 2600 eV.

Samples were air‐dried and ball‐milled to homogenise the sample and spread evenly onto adhesive carbon tape mounted to the stainless‐steel sample holder. Two replicate spectra were collected for each sample. To assist with peak fitting (see below), spectra were also collected for 11 standards, namely: iron sulfide [S^2−^], iron disulfide [S^1−^], L‐glutathione‐oxidised [RSSR], L‐methionine [RSR], L‐cysteine [RSH], DL‐methionine sulfoxide [RS(=O)R], sodium sulfite [SO_3_^2−^], taurine [RS(=O)_2_OH], L‐cysteic acid monohydrate [RS(=O)_2_O^−^], sodium dodecyl sulfate [ROSO_3_] and sodium sulfate [SO_4_^2−^](Table S1) (Prietzel et al. 2011, 2019). These compounds encompass a range of S oxidation states from −2 to +6. Standards were diluted to 1.5% S with boron nitride. The data for XANES spectra are available online (Barnard et al. 2024).

### 2.4 XANES Peak Fitting

Deconvolution of the S K‐edge XANES spectra was undertaken to quantify the relative proportions of five S species following Prietzel et al. (2003) and Prietzel et al. (2019). Five Gaussian curves were fit for the five S species: (1) Disulfide S (2472.6 eV), (2) Thiol and Thio‐ether (2474.2 eV), (3) Sulfinic acid and Sulfoxide S (2476.3 eV), (4) Sulfonate and Sulfonic acid (2481.0 eV), and (5) Sulfate‐S and Sulfate ester‐S (2482.7 eV) (Table S1). White‐line energies for the five Gaussian curves were verified using spectra collected from the 11 standards (Table S1). Spectra were first baseline corrected (from −52.25 to −15 eV) and edge normalized (normalization range + 40 to +100 eV). Replicate spectra were then merged to yield one spectrum per sample. Gaussian peak fitting was then undertaken using the peak fit function in Athena v. 0.9.26 (Ravel and Newville 2005). Suitable peak widths between 0 and 2 were first manually determined and then fixed for each peak (Table S2). No peaks were fit to reduced inorganic S forms (e.g., FeS or FeS_2_) ca. 2471–2472 eV or inorganic sulfite (SO_3_^2−^) ca. 2478 eV, as these were not detected in samples. Two additional arctangent functions were fit (fixed width of 0.3) to represent the edge steps of reduced S (2477 eV) and oxidised S (2483 eV). The proportion of each S species was calculated as the area under the curve for each peak over the sum of the area under the curve for all S peaks. Peak areas were multiplied by correction factors [Table S2, from Prietzel et al. (2019)] to account for the S oxidation state dependency of the absorption cross‐section.

### 2.5 Statistical Analysis

A principal component analysis (PCA) was undertaken on the total SOC and S as well as the proportion of S species derived from XANES using the factoextra and PCAtest packages in R (Camargo 2024; Kassambara and Fabian 2020; R Core Team 2023) to evaluate the interconnectivity of SOC, S, and S species across each of the agricultural experiments investigated.

## 3 Results

### 3.1 Changes to General Soil Properties

The Broadbalk Wheat Experiment allows for the assessment of soil properties during long‐term cropping for three different nutrient treatments. Total SOC and N contents differed between treatments. The FYM treatment consistently had the highest SOC across all time points (28 g kg^−1^ in 2010), followed by the NPKMgS treatment (10 g kg^−1^ in 2010), whilst the Control had the lowest SOC content (8.8 g kg^−1^ in 2010) (Table 1, Figure S3). In the case of the NPKMgS and Control, SOC contents fluctuated over time but generally remained stable; in contrast, SOC in the FYM treatment increased from 1865 (18 g kg^−1^) to 1914 (28 g kg^−1^) before remaining stable for the remainder of the period investigated. Total N followed a similar trend to SOC, although there was less difference between the Control (0.9 g kg^−1^ in 2010) and NPKMgS treatment (1.1 g kg^−1^ in 2010) while total N in FYM was 2.8 g kg^−1^ in 2010. The C:N ratios for the Broadbalk Wheat Experiment followed the trend: Control (9.1) ≈ NPKMgS (9.1) < FYM (10.3). Soil pH was neutral to alkaline across all treatments, although the Control plot was the most alkaline (pH ≈ 8, Table 1).

**TABLE 1:** Soil characteristic data for Broadbalk Wheat Experiment and the Wilderness sites.

Series	Plot	Year	Organic C (g C kg^−1^)	Total *N* (g N kg^−1^)	Total S (g S kg^−1^)	C:N	C:S	TES^1^ (g S kg^−1^)	EIS^2^ (g S kg^−1^)	EOS^3^ (g S kg^−1^)	pH (H_2_O)
Broadbalk Winter Wheat	Control	1865	9.4	1.1	0.18	9.0	54	0.036	0.036	0	—
		1914	8.6	1.0	0.15	8.8	56	0.022	0.020	0.0023	8.0
		1944	10	1.1	0.19	9.1	54	0.023	0.019	0.0043	8.0
		1987	9.8	1.1	0.18	9.1	55	0.020	0.015	0.0048	8.2
		2010	8.8	0.90	0.15	9.7	57	0.0061	0.0079	0	8.2
	FYM	1865	18	1.8	0.30	10	60	0.098	0.088	0.0098	7.5
		1914	28	2.7	0.46	10	61	0.047	0.031	0.016	7.4
		1944	25	2.3	0.39	11	63	0.037	0.026	0.011	7.6
		1987	28	2.8	0.52	10	55	0.038	0.026	0.012	7.8
		2010	28	2.8	0.45	10	62	0.016	0.012	0.0045	7.9
	NPKMgS	1865	ND	ND	0.22	—	—	0.037	0.031	0.0057	—
		1914	11	1.3	0.20	8.7	57	0.031	0.025	0.0060	7.8
		1944	11	1.3	0.27	8.9	41	0.066	0.063	0.0031	7.0
		1987	12	1.3	0.19	9.2	62	0.019	0.015	0.0035	7.7
		2010	10	1.1	0.18	9.5	58	0.011	0.011	0	7.2
Wilderness	Broadbalk	1881	9.2	1.0	0.17	8.8	56	0.019	0.015	0.0034	8.1
		1904	13	1.4	0.21	9.4	62	0.028	0.019	0.0086	8.0
		1964	27	2.6	0.39	11	70	0.040	0.030	0.0098	7.9
		1999	35	2.9	0.41	12	85	0.030	0.018	0.012	7.7
	Geescroft	1883	11	1.2	0.14	9.0	76	0.013	0.012	0.0013	7.1
		1904	14	1.3	0.18	10	75	0.024	0.020	0.0049	6.1
		1965	20	1.7	0.31	12	63	0.077	0.058	0.019	4.5
		1999	26	1.8	0.26	14	99	0.045	0.040	0.0056	4.4

*Note:* Organic C, total *N*, and soil pH are existing data sourced from Poulton et al. (2003) and the electronic Rothamsted Archive (Perryman 2015a, 2015b; Rothamsted Research 2018, 2021a). (1) Total extractable S, (2) Extractable inorganic S (SO_4_^2−^), (3) Extractable organic S.

Next, we assessed soil properties once cropped land was reverted to wilderness using the Broadbalk Wilderness and Geescroft Wilderness plots. The SOC and total N increased over time following rewilding, with SOC in the Broadbalk Wilderness plot increasing from 9.2 g kg^−1^ in 1881 to 35 g kg^−1^ in 1999, whilst the Geescroft Wilderness plot increased from 11 g kg^−1^ in 1883 to 26 g kg^−1^ in 1999 (Table 1, Figure S4). Total N in the two Wilderness plots followed the same trend as for SOC, increasing over time. The C:N ratios also increased over time for both Wilderness plots, from an average of 8.9 to 14 during the investigated period. Soil pH within the Broadbalk Wilderness plot remained neutral. In contrast, pH at the Geescroft Wilderness site was neutral at the first time point (1883) but became more acidic over time, with a final pH of 4.4 in 1999 (Table 1).

### 3.2 Changes to Total S and Extractable S Over Time

As expected, changes in total S reflected the changes in SOC contents. In the Winter Wheat Experiment, total S in the Control and NPKMgS treatments remained constant over time, with an average of 0.17 g kg^−1^ for the Control and 0.21 g kg^−1^ for the NPKMgS treatment. Total S in the FYM treatment fluctuated over time but generally increased from 0.3 g kg^−1^ in 1865 to 0.45 g kg^−1^ in 2010 (Table 1 and Figure S3). Similarly, total S in the Broadbalk Wilderness plot increased from 0.17 to 0.41 g kg^−1^ between 1881 and 1999, while Geescroft Wilderness increased from 0.14 to 0.26 g kg^−1^ between 1883 and 1999 (Table 1 and Figure S4).

Within the Broadbalk Winter Wheat Experiment, total extractable S had different trends for each treatment (Table 1, Figure S5). Total extractable S in the Control plot decreased steadily from 0.036 g kg^−1^ in 1865 to 0.0061 g kg^−1^ in 2010 and was primarily comprised of inorganic sulfate (> 76%). The NPKMgS treatment was generally similar to the Control with a decrease in total extractable S from 0.037 to 0.011 g kg^−1^ from 1865 to 2010, although it did increase temporarily in 1944 (0.066 g kg^−1^). Total extractable S in the FYM plot was consistently higher than the Control and NPKMgS plots but also decreased over time from 0.098 g kg^−1^ in 1865 to 0.016 g kg^−1^ in 2010. However, The FYM treatment had a higher proportion of extractable organic S compared to the other two treatments and fluctuated between 0.0045 and 0.016 g kg^−1^. In contrast, total extractable S at the rewilding sites showed an overall increase over time (Table 1 and Figure S6). The Broadbalk rewilding site had increased from 0.019 g kg^−1^ in 1881 to 0.030 g kg^−1^ in 1999, while the Geescroft rewilding site increased from 0.013 g kg^−1^ in 1883 to 0.045 kg^−1^ in 1999. The amount of extractable organic S increased over time in the Broadbalk rewilding site from 0.0034 g kg^−1^ to 0.012 g kg^−1^. Extractable organic S at the Geescroft rewilding site also increased from 0.0013 kg^−1^ to 0.0056 g kg^−1^ between 1883 and 1999.

### 3.3 Synchrotron‐Based XANES

**FIGURE 1:**
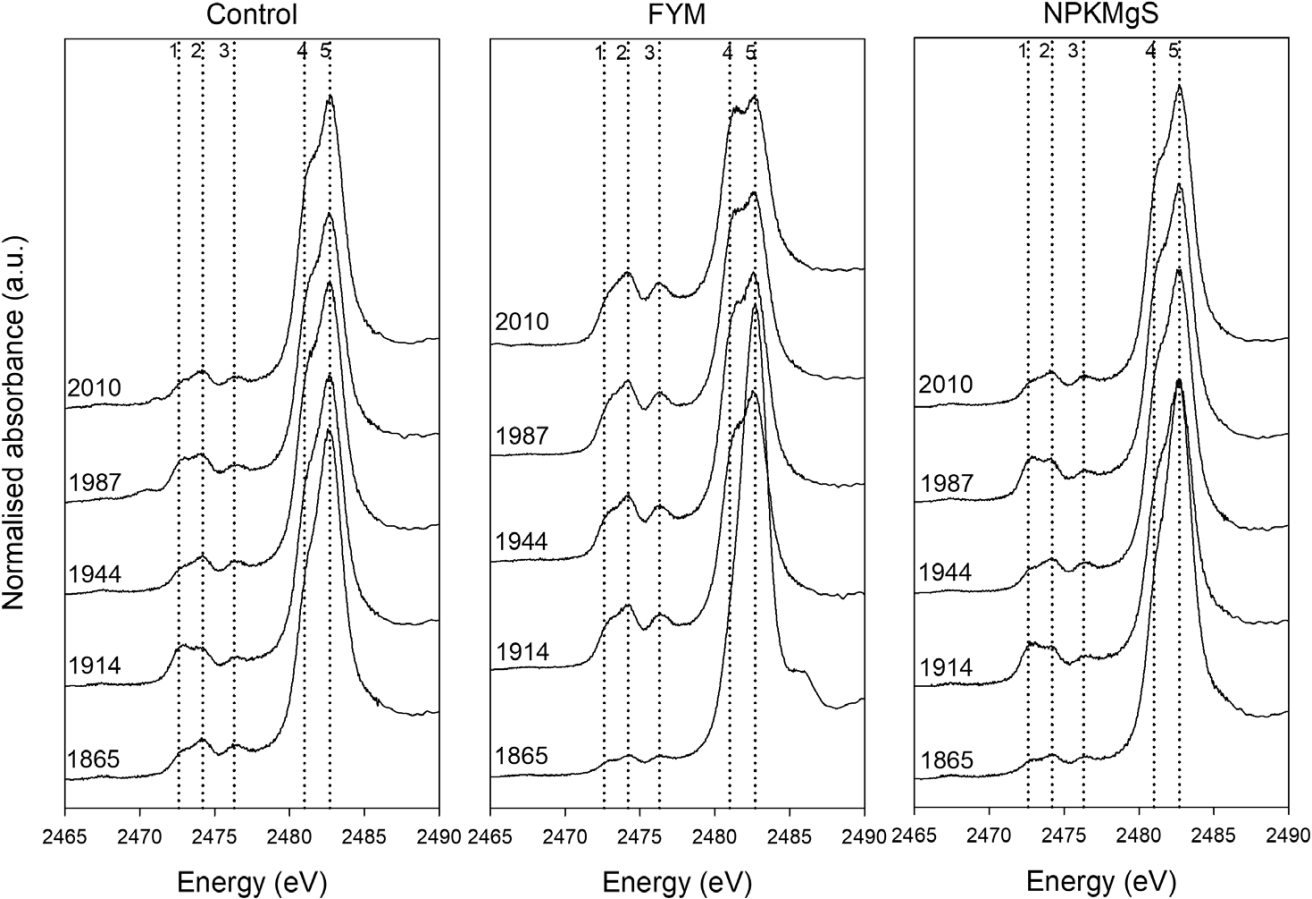
XANES spectra for the three contrasted treatments of the Broadbalk Wheat Experiment. The dotted vertical reference lines: (1) Disulfide S (2472.6 eV), (2) Reduce S forms, Thiol and Thio‐ether (2474.2 eV), (3) Sulfinic acid and Sulfoxide S (2476.3 eV), (4) Intermediate S forms, Sulfonate and Sulfonic acid (2481.0 eV), and (5) Oxidised S forms, Sulfate‐S and Sulfate ester‐S (2482.7 eV).

**FIGURE 2:**
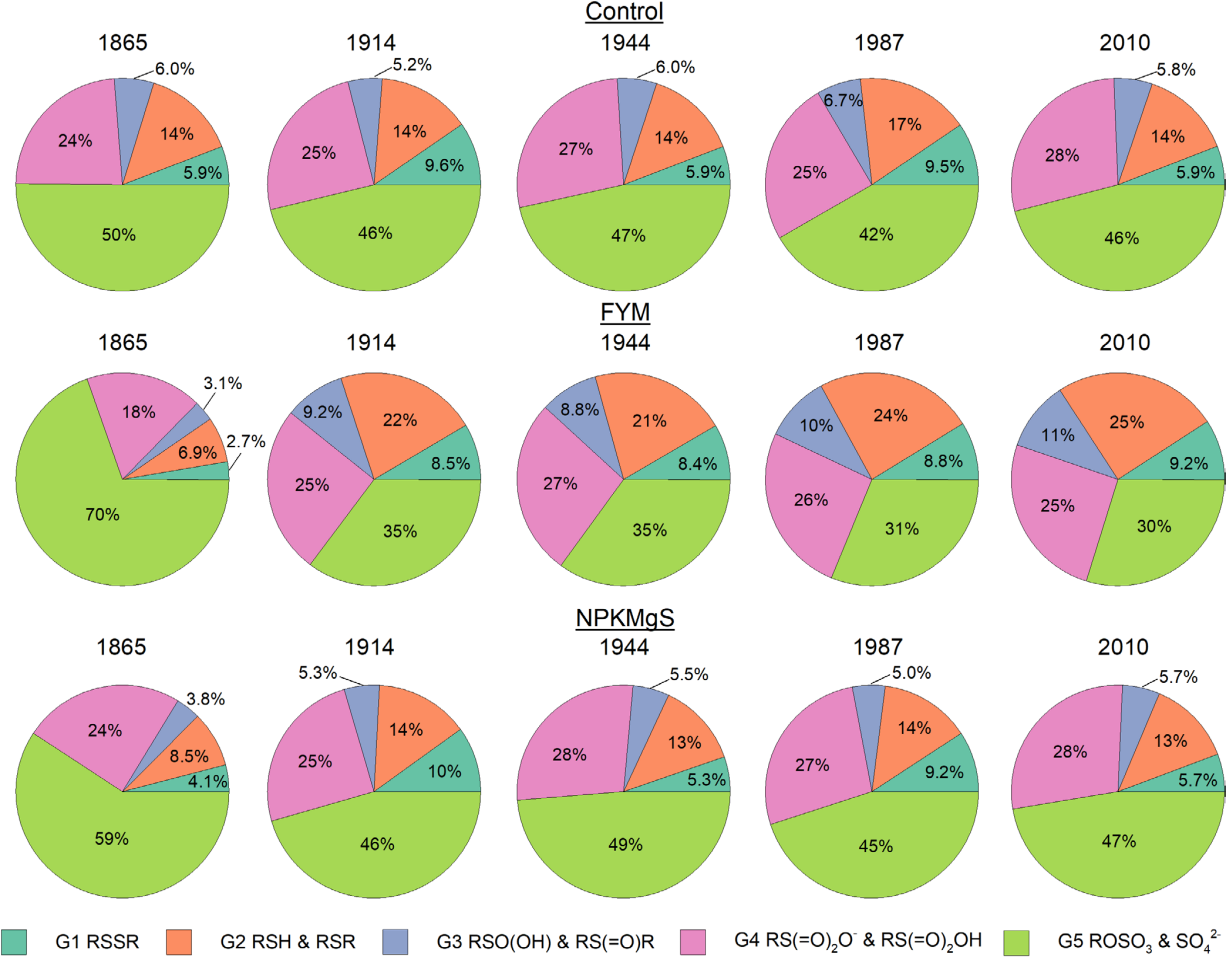
Relative proportions (%) of S species groups over time (1865, 1914, 1944, 1987, and 2010) for all three treatments of the Broadbalk Wheat Experiment. Five S species were identified: (G1) Disulfide S (2472.6 eV), (G2) Thiol and Thio‐ether (2474.2 eV), (G3) Sulfinic acid and Sulfoxide S (2476.3 eV), (G4) Sulfonate and Sulfonic acid (2481.0 eV), and (G5) Sulfate‐S and Sulfate ester‐S (2482.7 eV).

We examined the spectra from the soils collected from the Broadbalk Wheat Experiment to assess changes in S speciation in soil caused by different nutrient treatments during long‐term cropping (Figures 1 and 2). When comparing the three treatments at the final time point (2010), it was found that the Control and NPKMgS were similar, whilst the FYM had increased proportions of reduced S. For example, for the Control and NPKMgS treatment, the two most dominant forms of S were the intermediate forms of S (i.e., G4: sulfonate S and sulfonic acid, +4 to +5, accounting for 28% of S species) and oxidised forms of S (i.e., G5: organic and inorganic sulfate S, +6, accounting for 47% of S species). In contrast, the more reduced forms of S accounted for a higher proportion of the S species in the FYM treatment, with G2 (RSH and RSR, +0.5) accounting for 25% of the S species (c.f. an average of 14% in the Control and NPKMgS treatments). Indeed, it was noted that C‐bonded S (i.e., the more reduced forms of S, G1‐4) was higher for FYM (70%) than for the Control (54%) and NPKMgS treatments (53%) (Table S3).

Next, we examined trends in S speciation over time for the Broadbalk Wheat Experiment (Figure 2). For both the Control and the NPKMgS treatments, changes over time were comparatively small, with the proportion of oxidised S (G5: organic and inorganic sulfate S, +6) varying from an average of 55% in 1865 to 47% in 2010, while the proportion of intermediate S (G4: sulfonate S and sulfonic acid, +4 to +5) fluctuated between 24% and 28%. Again, the FYM treatment was most distinct, having the largest change over time from 1865 to 2010, with oxidised S (organic and inorganic sulfate S, +6) decreasing from 70% to 30%, whilst there was an overall increase in all forms of C‐bonded S (G1‐4) from 30% in 1865 to 70% in 2010 (Table S3).

**FIGURE 3:**
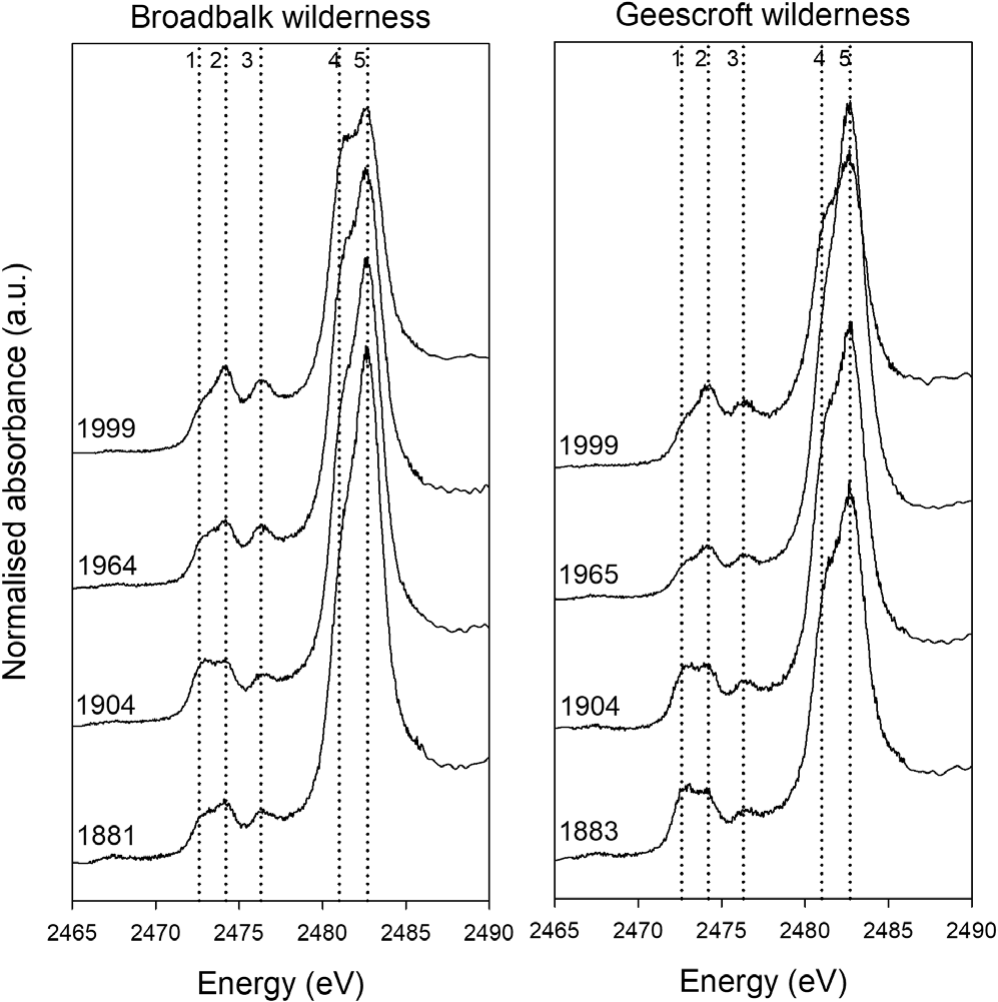
XANES spectra for the Broadbalk and Geescroft Wilderness plots. The dotted vertical reference lines: (1) Disulfide S (2472.6 eV), (2) Reduce S forms, Thiol and Thio‐ether (2474.2 eV), (3) Sulfinic acid and Sulfoxide S (2476.3 eV), (4) Intermediate S forms, Sulfonate and Sulfonic acid (2481.0 eV), and (5) Oxidised S forms, Sulfate‐S and Sulfate ester‐S (2482.7 eV).

**FIGURE 4:**
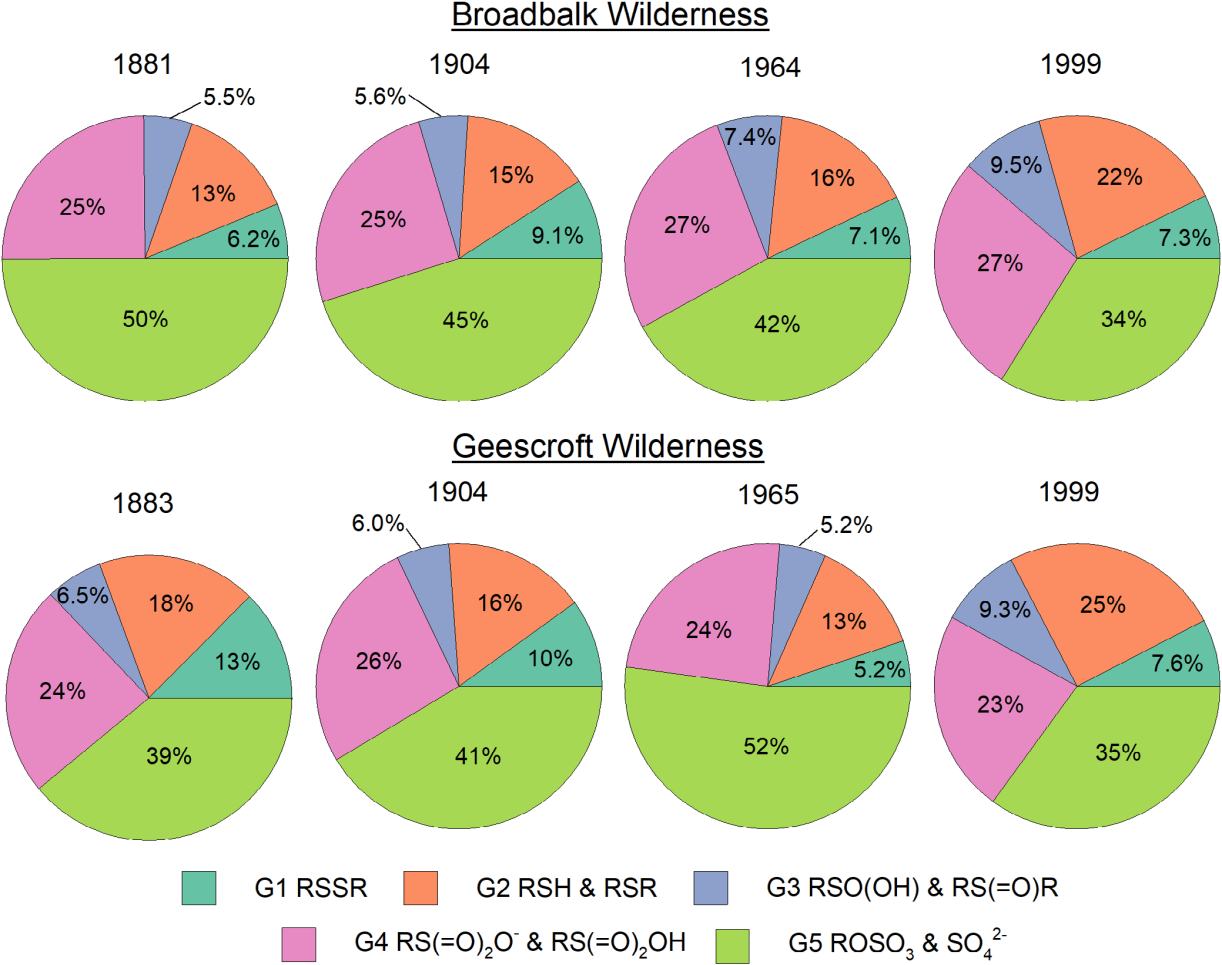
Relative proportions (%) of S species groups over time in the Broadbalk Wilderness plot (1881, 1901, 1964, and 1999) and Geescroft Wilderness plot (1883, 1904, 1965, and 1999). Five S species were identified: (G1) Disulfide S (2472.6 eV), (G2) Thiol and Thio‐ether (2474.2 eV), (G3) Sulfinic acid and Sulfoxide S (2476.3 eV), (G4) Sulfonate and Sulfonic acid (2481.0 eV), and (G5) Sulfate‐S and Sulfate ester‐S (2482.7 eV).

The effect of rewilding on S speciation was assessed using the Broadbalk and Geescroft Wilderness sites (Figure 3). Clear trends were evident over time from the commencement of wilderness regeneration (Figure 4). Specifically, at the beginning of regeneration at the Broadbalk site, the proportion of oxidised S (G5: organic and inorganic sulfate S, +6) decreased from 50% in 1883 to 34% in 1999, while C‐bonded S (G1‐4) increased from 50% in 1883 to 66% in 1999 (Table S3). In contrast, from 1883 to 1965, oxidised S (G5: organic and inorganic sulfate S, +6) increased over time for the Geescroft Wilderness site following the commencement of wilderness regeneration and a simultaneous decrease in the most reduced forms of S (G1: disulfide S, +0.2 and G2: thiol and thio‐ether S, +0.5). However, the reverse occurred between 1965 and 1999, with an abrupt decrease in oxidised S (G5: organic and inorganic sulfate S, +5) from 52% to 35% in conjunction with an increase in G2 (thiol and thio‐ether S, +0.5) from 13% to 25% (Table S3).

Extractable S contents (Table 1) and deconvolution data (Table S3) were then combined to distinguish between inorganic sulfate S [SO_4_^2−^] and organic sulfate ester‐S [ROSO_3_] (Table 2) in a similar approach as described by Prietzel et al. (2013). Within the Control and NPKMgS treatments, the proportion of inorganic sulfate decreased over time; however, no clear trends were observed for the proportion of sulfate ester‐S. For the FYM treatment, the proportions of both inorganic sulfate and sulfate ester‐S decreased. As a result, the C‐bonded S:sulfate ester‐S ratio steadily increased over the cropping period from 0.74 to 2.6. The Broadbalk rewilding site displayed similar trends to the FYM treatment—with both inorganic sulfate and sulfate ester‐S decreasing combined with an increasing C‐bonded S:sulfate ester‐S ratio. In contrast, the Geescroft rewilding site generally displayed an increase in the proportion of inorganic S, with sulfate ester‐S decreasing. The C‐bonded S:sulfate ester‐S ratio fluctuated, decreasing from 2.0 in 1883 to 1.4 in 1965 before increasing to 3.3 in 1999.

**TABLE 2:** Proportion (%) of total S that is sulfate‐S or sulfate ester‐S estimated for the bulk soil.

Series	Plot	Year	Relative proportion (%)			C‐bonded S:sulfate ester‐S
			Inorganic sulfate and sulfate esters [SO_4_^2−^ & ROSO_3_]^1^	Inorganic sulfate [SO_4_^2−^]^2^	Sulfate ester [ROSO_3_]^3^	
Broadbalk winter wheat	Control	1865	50	20	30	1.7
		1914	46	13	33	1.6
		1944	47	10	37	1.4
		1987	42	8.6	33	1.7
		2010	46	5.1	41	1.3
	FYM	1865	70	30	40	0.74
		1914	35	6.8	28	2.3
		1944	35	6.6	28	2.3
		1987	31	4.9	26	2.6
		2010	30	2.6	27	2.6
	NPKMgS	1865	59	14	45	0.92
		1914	46	12	34	1.6
		1944	49	23	26	2.0
		1987	45	7.9	37	1.5
		2010	47	6.3	41	1.3
Wilderness	Broadbalk	1881	50	9.2	41	1.2
		1904	45	9.0	36	1.5
		1964	42	7.8	34	1.7
		1999	34	4.4	30	2.2
	Geescroft	1883	39	8.2	31	2.0
		1904	41	11	30	1.9
		1965	52	18	34	1.4
		1999	35	15	20	3.3

*Note:* (1) Determined from x‐ray absorption near‐edge structure (XANES) spectroscopy, (2) determined from ion chromatography (IC), (3) determined by difference (Prietzel et al. 2013).

**FIGURE 5:**
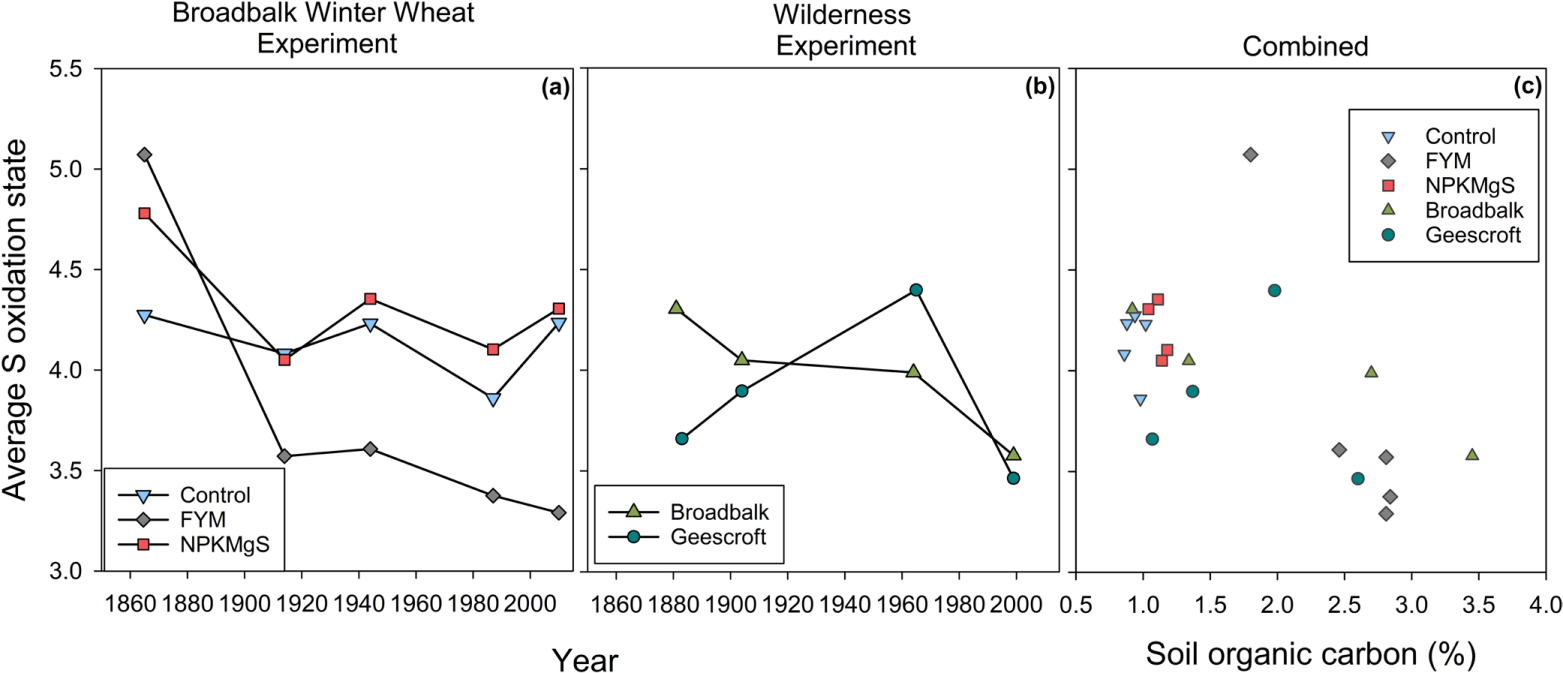
Weighted average of the oxidation state of S across time under long‐term cropping (a, Broadbalk Winter Wheat Experiment for three nutrient treatments) and rewilding (b, Broadbalk Wilderness and Geescroft Wilderness Experiments), and (c) the weighted average of the oxidation state of S versus SOC at the Broadbalk Winter Wheat Experiment and the Broadbalk and Geescroft Wilderness Experiments.

Finally, we assessed changes to the average S oxidation state by calculating the weighted average of the oxidation states relative to the proportion of each S species within the sample in a similar approach to Prietzel et al. (2007). Under long‐term cropping (Figure 5a) the average oxidation state within the Control and NPKMgS treatments generally remained stable. In contrast, the FYM treatment had a decrease in the average S oxidation state, decreasing from +5.1 in 1865 to +3.3 in 2010. For the rewilding sites (Figure 5b), the average oxidation state for the Broadbalk Wilderness decreased steadily over time (from +4.3 in 1881 to +3.6 in 1999), whilst the Geescroft Wilderness site had no clear trend. Moreover, increasing SOC content was generally associated with a decline in the average oxidation state of S (Figure 5c).

### 3.4 Relationship Between SOC and Soil S Properties

**FIGURE 6:**
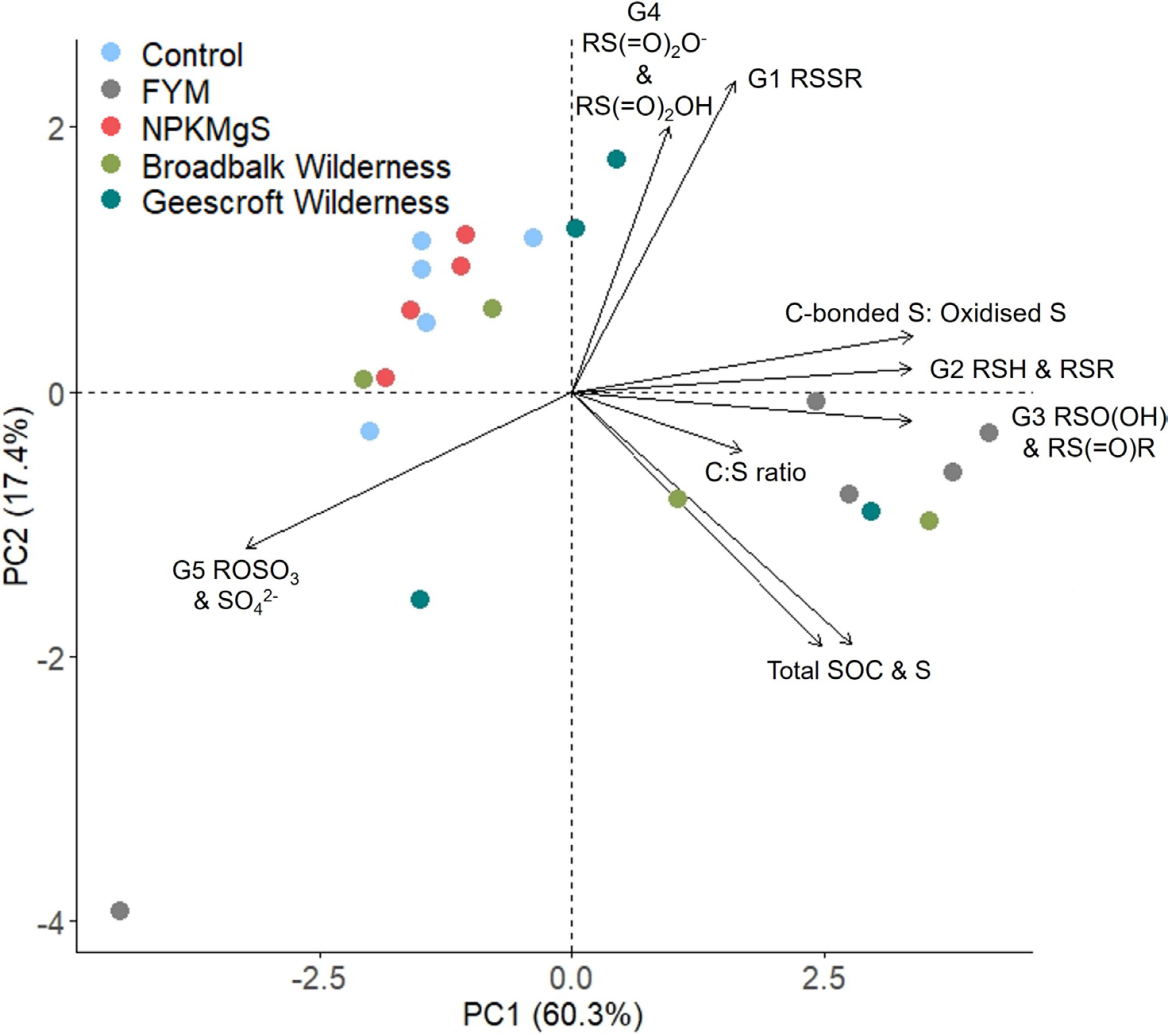
Principal component analysis biplot visualising the variation in total SOC, total S, and C:S ratio in relation to S species (G1 Disulfide S, G2 Thiol and Thio‐ether, G3 Sulfinic acid and Sulfoxide S, G4 Sulfonate and Sulfonic acid, and G5 Sulfate‐S and Sulfate ester‐S) across all agricultural experiments investigated. Arrows indicate the loadings of each variable. A matrix of principal components is presented in Table S4.

Principal component analysis (Figure 6) enabled the interconnectivity of SOC and soil S properties to be evaluated when results from all agricultural trials were combined. The first component (PC1) relates to the inverse relationship between oxidised S (G5) and other soil properties examined. The second component (PC2) relates to the weakly inverse relationship between the most reduced forms of C‐bonded S (G1) and intermediate S (G4) and the total SOC and total S. The PCA biplot reveals the similarities between the Control and the NPKMgS treatments, with these being distinct from the FYM treatment. The Geescroft and Broadbalk Wilderness sites were the most variable as a result of the more prominent changes in SOC contents and S speciation over time during rewilding.

## 4 Discussion

### 4.1 Effect of Long‐Term Cropping on S Speciation

The different nutrient treatments changed S speciation in this long‐term cropping system. Soil S was predominantly in intermediate and oxidized states where either inorganic NPKMgS or no amendments (Control) were applied. For these treatments, S speciation remained stable over time with minimal changes to the average oxidation state of S (Figures 2 and 5a). In contrast, the application of organic amendment in the form of FYM had a pronounced impact on S speciation, with an increased proportion of C‐bonded S over time (Table 2 and Table S3) and, notably, more reduced forms of S (Figure 2), which were also evidenced by the decrease in the average oxidation state over time (Figure 5a). Our findings are similar to those reported by Xu et al. (2016), who assessed the influence of 21 years of manure and NPK mineral fertilizer additions on S speciation in soils in different climatic zones in China. The authors reported soil amendments had a distinct influence on S speciation regardless of soil type, with FYM application resulting in increased reduced and intermediate forms of S. In contrast, S speciation under NPK mineral fertilizer was dominated by oxidized S (Xu et al. 2016).

The changes in S speciation observed in the Broadbalk Winter Wheat experiment (Figures 1 and 2) are related to the changes in SOC and total S. Specifically, the addition of FYM increased SOC from 18 to 28 g kg^−1^ (Table 1, Figure S3), which is comparable to the increase in SOC observed at the Wilderness sites. As expected, this increase in SOC translated to an increase in organic sources of S (Förster et al. 2012), subsequently resulting in a concomitant increase in reduced C‐bonded S (Figure 2) since organic S is known to be dominant in more reduced forms of C‐bonded S such as amino acids, cysteine, and methionine (Scherer 2009). Indeed, C‐bonded S in the FYM treatment increased from 30% in 1865 to 70% of S species in 2010 (Table S3). In contrast to FYM, SOC concentrations in the NPKMgS and Control treatments were lower (~9.4–11 g kg^−1^) and remained relatively constant over the 145‐y experimental period (Table 1, Figure S3). As a result, more oxidised forms of S were dominant in the NPKMgS and Control treatments; for example, in 2010, organic and inorganic sulfate S [G5] accounted for 30% of all S species in the FYM treatment, but 47% in the NPKMgS and Control treatments, and like SOC for the NPKMgS and Control treatments, remained relatively constant over time (Figure 2). Our observations that increasing SOC through FYM applications tended to increase the proportion of more reduced forms of C‐bonded S across 145 years align with previous studies such as Prietzel et al. (2007) and Castellano and Dick (1988). However, our finding that S speciation remained relatively constant over 145 years in the NPKMgS and Control treatments and that no change in the average oxidation state of S was observed (Figure 5a,c) was somewhat unexpected given that cropping and associated soil disturbance have been shown to shift S towards more oxidised forms (Siebers and Kruse 2019; Solomon et al. 2011, 2003). However, cropping in these previous studies was also associated with a marked decrease in SOC (e.g., in the studies of Solomon et al. (2003), SOC decreased from ~90 to 38 g kg^−1^). In contrast, in the present study, SOC and total S were comparatively stable in the NPKMgS and Control treatments. This is likely due to SOC having already reached a state of equilibrium by 1865 (the first sampling time in the present study) (Murty et al. 2002), given that arable cropping had occurred for centuries before the start of the experiment (Macdonald et al. 2018). Interestingly, despite FYM application resulting in contrasting trends in SOC contents and S speciation compared to the Control and NPKMgS treatments, the amount of plant available S (extractable inorganic S) decreased in all three treatments over time (Table 1 and Figure S5). This highlights the net loss of plant‐available S under cropping, through leaching and/or removal by crops (Knights et al. 2000), regardless of the amendments applied to the soil.

The different organic S forms reflect different mineralisation pathways, with C‐bonded S (XANES peaks G1‐G4) resulting from in vivo biological mineralisation and being regulated by microbial demand for energy, while sulfate ester‐S is derived from ex vivo biochemical processes regulated by the microbial demand for S (McGill and Cole 1981; Scherer 2009; Solomon et al. 2003). The C‐bonded S:sulfate ester‐S ratio, therefore, serves as an indicator of the source of organic S mineralisation (Malik et al. 2020). In the Control and NPKMgS treatments, the C‐bonded S:sulfate ester‐S ratio fluctuated but did not consistently change over time (Table 2), suggesting a balance between biological and biochemical mineralisation. This contrasts with the findings of Solomon et al. (2005), who reported a proportionately larger loss of sulfate ester‐S through biochemical mineralisation when cropping exceeded 20 years. However, this finding in the present study is likely also attributed to the relatively stable SOC and S concentrations over time in these treatments (Table 1, Figure S3). Both inorganic sulfate and sulfate ester‐S decreased over time in the FYM treatment, which resulted in a steady increase in the C‐bonded S:sulfate ester‐S ratio. While C‐bonded S is often regarded as the most easily mineralisable source of S during land disturbance (Churka Blum et al. 2013; Ma et al. 2021; Solomon et al. 2011), the FYM amendments may have continually replenished any C‐bonded S mineralised, thereby preventing or compensating for a proportionately greater loss of C‐bonded S relative to sulfate ester‐S.

The long‐term assessment of S speciation under cropping for 145 years also sheds light on the relative sensitivities of different S forms. The most reduced forms of S (G1: disulfide, and especially G2: thio‐ether and thiol ‐S) and oxidised S (G5: sulfate and sulfate ester‐S) were the most sensitive, displaying the most distinct changes between different treatments across the studied period. In contrast, the changes to intermediate forms of S (G3 and G4: sulfinic and sulfonic acid, and sulfoxide and sulfonate S) were comparatively minor (Table S3). Similar trends for intermediate forms of S were also found in soils from different land uses in Ethiopia by Solomon et al. (2003). Schroth et al. (2007) postulated that this could either demonstrate the less labile nature of intermediate S or reflect the transient nature of intermediate S as the reduced forms of S are mineralized to oxidized S and vice versa. In this case, intermediate S is most likely the transition point for S mineralization as changes in the proportion of reduced S forms are mirrored by the changes in oxidized S forms (Figure 2).

**FIGURE 7:**
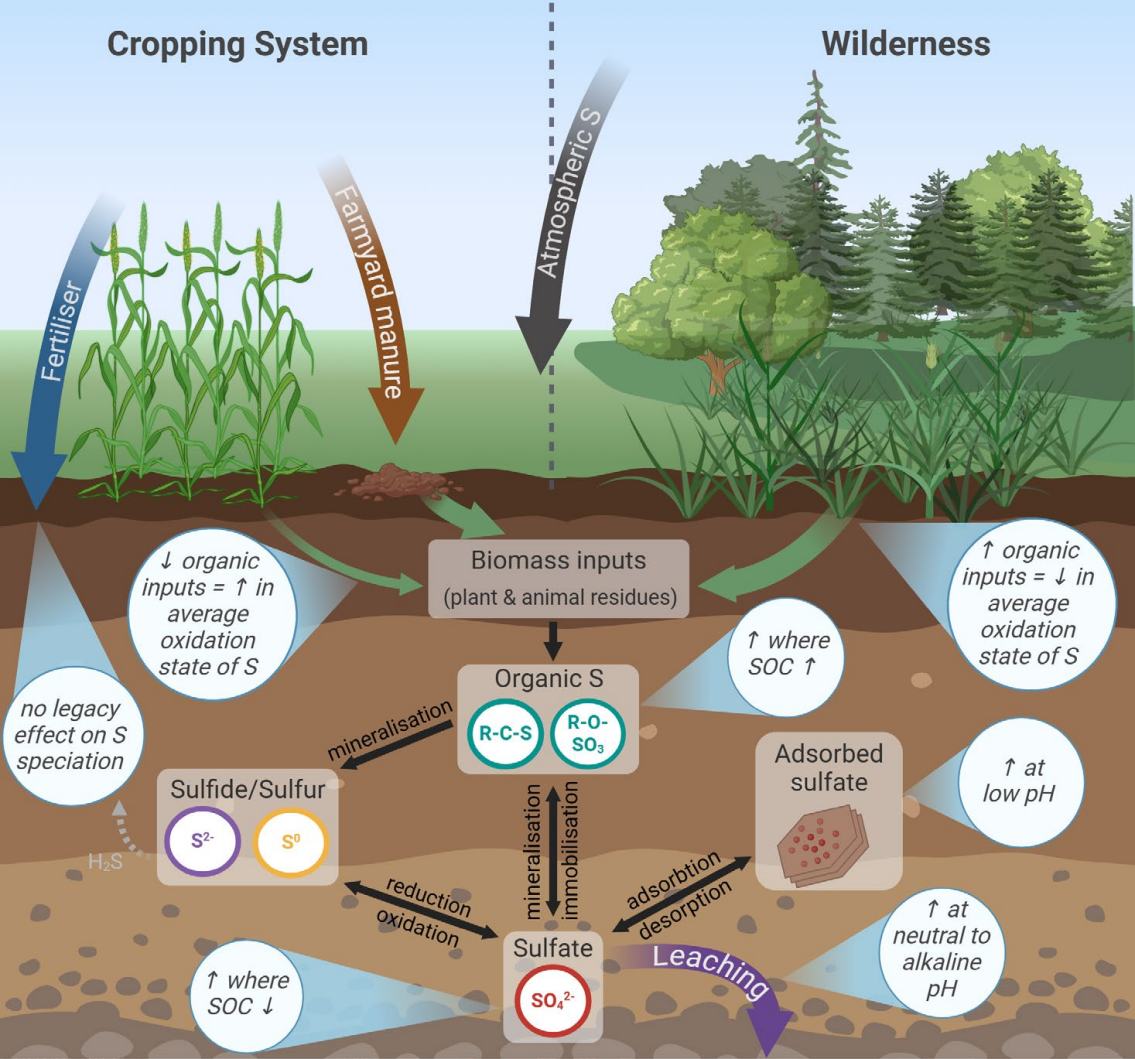
A conceptual diagram examining changes in S speciation in the soil S cycle, based on long‐term trends from the Broadbalk Winter Wheat Experiment and the Broadbalk and Geescroft Wilderness sites.

The similarities in S speciation of the Control and NPKMgS treatments revealed by XANES support earlier findings by Knights et al. (2001) that decades of S mineral fertiliser application have not had a legacy effect on S speciation. Given the comparatively low removal of S in grain relative to S applied by fertiliser, this finding in the present study can most likely be attributed to the highly mobile nature and subsequent sulfate leaching (Förster et al. 2012; Tabatabai 1987). The same result had been observed by Costa et al. (2022) and Costa and Crusciol (2016) and was predominantly attributed to the higher pH and greater SOC contents within surface soils, creating a net negative mineral surface charge, facilitating the leaching of sulfate. Given the neutral to alkaline soil pH at the Broadbalk Winter Wheat Experiment, the surface charge of soil minerals was most likely unfavourable for sulfate adsorption (Figure 7). Indeed, evidence from a one‐year study reporting sulfate concentrations in drainage waters collected from an adjacent NPKMgS plot receiving equivalent S inputs (plot 7—the current study investigates plot 8) revealed substantially higher sulfate concentrations in drainage waters from the NPKMgS plot (30–40 mg^—^sulfate‐S L^−1^) compared to both the Control and FYM plots (< 10 mg^—^sulfate‐S L^−1^) (Knights et al. 2000).

### 4.2 Effect of Conversion of Cropped Land to Wilderness on S Speciation

The two Wilderness sites were used to investigate changes in S speciation following the regeneration of cropped land to wilderness. Interestingly, despite both plots being converted to similar land uses and consistently accumulating SOC content over time (Table 1), the changes in S speciation differed markedly between the two sites (Figure 4 and Figure 5b). The Broadbalk Wilderness site responded as expected—the gradual accumulation of SOC and total S during rewilding (Table 1, Figure S4) resulted in an increased dominance of reduced C‐bonded S forms (Figure 2) and a decrease in the average oxidation state of S (Figure 5b), with similar results reported by Prietzel et al. (2007). However, for the Geescroft Wilderness site, trends in S speciation preceding 1965 were unexpected, with a pronounced increase in oxidised S (Figures 4 and 5b) despite increasing SOC (Table 1).

Unlike the Broadbalk Wilderness site, which had been heavily limed (Poulton et al. 2003), Geescroft Wilderness remained unlimed and has thus shown a substantial decrease in soil pH from 7.1 in 1883 to 4.4 in 2010 (Table 1) (Macdonald et al. 2018). We suggest that this soil acidification in the Geescroft Wilderness may have increased sorption and, therefore, retention of sulfate anions, contributing to the pronounced increase in oxidised S (Figure 7). Indeed, sulfate retention increases considerably when soil pH < 6 due to the protonation and subsequent creation of a net positive charge on mineral surfaces (Edwards 1998; Tabatabai 1987). Similar findings were also made by Wang et al. (2024) when comparing long‐term (82 years) fertilised grassland sites with and without lime application in Germany.

A second factor contributing to the trends observed at the Geescroft site is that these acidic conditions created a favorable environment for retaining the increased quantities of S derived from rising atmospheric emissions preceding the 1980s (Zhao et al. 2003). Between 1965 and 1999, however, the expected increase in reduced C‐bonded S was observed (Figures 4 and 5b) as SOC content continued to increase while pH had primarily remained unchanged (Table 1) and the atmospheric deposition of S declined (Zhao et al. 2003). This finding provides interesting insights into the relative control of SOC content and soil pH on S speciation and can be related to previous findings by Boye et al. (2011). The authors found contrasting S speciation in three soils after the application of FYM—one soil displayed a shift to intermediate forms of S, the second had no clear trend, while a shift to oxidized S forms was reported for the third. Inspection of their soil chemical data shows the trend in S speciation closely follows a decrease in soil pH across the three soils—that is, the soil with the greatest proportion of reduced S had the highest pH (pH = 7.3) while the soil with the greatest proportion of oxidized S had the lowest pH (pH = 5.6). Thus, perhaps the findings by Boye et al. (2011) combined with the present study's findings indicate the potential for soil chemical properties, such as pH, to reduce the influence of SOC content on S speciation. However, we suggest additional investigation is required to confirm the role of soil pH in soil S speciation. Finally, after 1965, there was no further change in pH in the soil at the Geescroft Wilderness site; hence, no further increase in negative charge sites occurred, but there was a substantial increase in SOC; hence, an increase in reduced C‐bonded S (Figure 7).

### 4.3 Broader Implications for S Cycling and Global S Management

Our study demonstrates the long‐term changes in the stoichiometry of C and S as well as changes in the relative abundance of the most reduced C‐bonded S (namely thiol and thio‐ether) and oxidised S (sulfate ester‐S and sulfate‐S), thereby highlighting the interconnectivity of SOC and S (Figure 6). Moreover, these findings highlight the importance of several mechanisms involved in the broader formation, stability, and turnover of soil organic S, especially regarding anthropogenic changes in key components of the S cycle (Figure 7). For example, our results demonstrate that factors influencing SOC (such as changes in biomass inputs, or increased SOC mineralisation) also result in concomitant changes in organic S. Not only did land‐use change alter the turnover of soil organic S (and SOC), but our results also demonstrated that changes in land‐use alter the different forms of organic S, especially sulfate esters (which are generally considered more labile) and C‐bonded S (which are generally considered more stable). As expected, greater SOC contents coincide with higher total S (Kumar et al. 2022), although this is also coupled with comparatively higher C:S ratios and more reduced C‐bonded S. The converse is found for lower SOC environments with comparatively greater proportions of oxidised S, particularly sulfate ester‐S (Figure 6 and Table 2). This finding highlights the beneficial and lasting impact of organic amendments such as FYM, not only for increasing total SOC and S but also for maintaining the abundance of S species containing thiol and thio‐ether functional groups (soil S sinks). These S species are most readily utilised by microbes (Ma et al. 2021; Wang et al. 2024), which may in turn support the slow‐release of plant‐available S.

With S deficiency becoming increasingly problematic (Zenda et al. 2021), researchers have emphasized the need for adequate S management strategies in agricultural soils that do not necessarily rely solely on the use of mineral fertilizers (Gerson and Hinckley 2023). In light of this, the trends observed in the current study demonstrate the long‐term impacts of different management practices on soil S speciation, that is, no fertilizer, inorganic mineral fertilizer, organic amendment, and rewilding. This can guide land management strategies from the perspective of sustaining S availability. Thus, practices that promote organic matter accumulation, such as reduced tillage and residue retention (Li et al. 2020), can be prioritized. Specifically, the increases in reduced C‐bonded S with farmyard FYM applications highlight its role in enhancing reduced organic S, which can be a slow‐release S source for crops. This aligns with findings by Suran et al. (2023) demonstrating organic amendments such as FYM were the best strategy to maintain soil S long‐term compared to the application of mineral S. Furthermore, the limited retention of oxidized S in soils treated with NPKMgS fertilizers suggests a need for balanced fertilizer strategies that maintain long‐term S availability while preventing leaching losses of sulfate. This is particularly apparent for neutral to alkaline soils where sulfate is more susceptible to leaching (Figure 7)—as demonstrated by findings of the current study and previous studies undertaken at Rothamsted (Knights et al. 2000). Thus, the key findings gained from the current study (Figure 7) can be used to develop predictive models for S speciation under different land‐use and amendment scenarios, aiding in the design of adaptive management strategies.

## 5 Conclusion

This long‐term study of the Broadbalk Wheat Experiment reveals insights into S speciation dynamics in agricultural soils. Over 145 years, organic amendments (FYM) altered soil S speciation, increasing C‐bonded S and reducing the average oxidation state of S. The S speciation in the inorganic fertilizers (NPKMgS) and unamended Control treatments predominated in oxidised S and remained unchanged. These trends closely relate to SOC content, highlighting the interconnectivity of SOC and S cycling in soils. From the Broadbalk and Geescroft Wilderness sites, it was clear that soil pH is a critical factor influencing S speciation together with SOC accumulation—soil acidification at Geescroft Wilderness initially increased proportions of oxidised S forms despite increasing SOC. This observation contrasted with the expected trend in S speciation that was observed at Broadbalk Wilderness. These findings provide new information on the factors that control the speciation and availability of S in agricultural soils.

## Author Contributions

**Meghan Barnard:** formal analysis, investigation, methodology, writing – original draft, writing – review and editing. **Brigid A. McKenna:** conceptualization, data curation, formal analysis, investigation, writing – original draft, writing – review and editing. **Ram C. Dalal:** conceptualization, formal analysis, writing – original draft, writing – review and editing. **Steve P. McGrath:** investigation, methodology, visualization, writing – original draft, writing – review and editing. **Zhe H. Weng:** project administration, writing – review and editing. **Jeremy L. Wykes:** formal analysis, investigation, methodology, writing – review and editing. **Peter M. Kopittke:** conceptualization, formal analysis, investigation, methodology, writing – original draft, writing – review and editing.

## Conflicts of Interest

The authors declare no conflicts of interest.

## Supporting information


Data S1.

## Data Availability

The data that support the findings of this study are openly available in The University of Queensland. Data Collectionat https://doi.org/10.48610/9773a9d. Total organic C and total N values were sourced from the electronic Rothamsted Archive at https://doi.org/10.23637/KeyRefOABKWoc, https://doi.org/10.23637/KeyRefOAGEWoc, https://doi.org/10.23637/BK‐oadata‐soilN‐01, and https://doi.org/10.23637/KeyRefOABKsoc‐02.
