# Supplementary material for: Sulfur's Long Game: 145 Years of Soil Sulfur Speciation in the World's Oldest Agricultural Experiments
Source: Glob Chang Biol. 2025 Mar 20;31(3):e70136. doi: 10.1111/gcb.70136 (PMC11924157; doi:10.1111/gcb.70136)
Supplement: Supplementary file 1 — Data S1. [file GCB-31-e70136-s001.pdf]

Sulfur's Long Game: 145 years of soil sulfur speciation in the world's oldest agricultural experiments

Running title: Sulfur speciation after land use change

Meghan Barnard[1], Brigid A. McKenna[1], Ram C. Dalal[1], Steve P. McGrath[2], Zhe H. Weng[3], Jeremy L. Wykes[4], Peter M. Kopittke[1*]

[1] *The University of Queensland, School of Agriculture and Food Sustainability, St Lucia, Queensland, 4072, Australia*

[2] *Rothamsted Research, West Common, Harpenden AL5 2JQ, United Kingdom*

[3] *The University of Adelaide, School of Agriculture, Food, and Wine, Urrbrae, South Australia 5064, Australia*

[4] *The Australian Synchrotron, Clayton, Victoria, 3168, Australia*

[*] Email: p.kopittke@uq.edu.au, Phone: +61 7 3346 9149

m.barnard@uq.edu.au, b.mckenna1@uq.edu.au, r.dalal@uq.edu.au, steve.mcgrath@rothamsted.ac.uk, han.weng@adelaide.edu.au, jeremyw@ansto.gov.a, p.kopittke@uq.edu.au.

Keywords: Soil sulfur, Broadbalk Winter Wheat Experiment, wilderness regeneration, farmyard manure, organic amendment, land management.

Type of paper: Primary research article

Supplementary Table S1 Sulfur standards used and the energy corresponding to the white-line peak

Standard	Valence	Functional group	Structure	White-line energy (eV)
Iron sulfide	-2	Inorganic sulfide	S^{2-}	2471.0
Iron disulfide	-1	Inorganic disulfide	S^{1-}	2472.0
L-glutathione oxidised[x]	+0.2	Disulfide	RSSR	(1) 2472.8
				(2) 2474.3
L-methionine	+0.5	Thio-ether	RSR	2473.6
L-cysteine	+0.5	Thiol	RSH	2473.4
DL methanione sulfoxide	+2	Sulfoxide	RS(=O)R	2476.3
Sodium sulfite	+4	Inorganic sulfite	SO_3^{2-}	2478.5
Taurine	+4	Sulfonic acid	$RS(=O)_2OH$	2481.1
L-Cysteic acid monohydrate	+5	Sulfonate	$RS(=O)_2O^-$	2481.2
Sodium dodecyl sulfate	+6	O-linked sulfate ester	$ROSO_3$	2482.7
Sodium sulfate	+6	Inorganic sulfate	SO_4^{2-}	2482.7

[x] – compound has two bonding environments (1s → σ*(S-S) and 1s → σ*(S-C))

Supplementary Table S2 XANES spectra peak fitting parameters

Peak	Peak Assignment (eV)	Peak width	Oxidation state of S	Species	Structure	Peak area correction factor
G1	2472.6	0.59	+0.2	Disulfide S	RSSR	0.9
G2	2474.2	0.72	+0.5	Thiol<br>Thio-ether	RSH<br>RSR	0.9
G3	2476.3	0.64	+2	Sulfinic acid<br>Sulfoxide S	RSO(OH)<br>RS(=O)R	0.6
G4	2481.0	0.77	+4 to 5	Sulfonate S<br>Sulfonic acid	$RS(=O)_2O^-$<br>$RS(=O)_2OH$	0.4
G5	2482.7	0.81	+6	Sulfate S<br>Sulfate ester	SO_4^{2-}<br>$ROSO_3$	0.35

Supplementary Table S3 Relative proportions (%) of each Gaussian peak. G1: Disulfide S (2472.6 eV), G2: Reduce S forms, Thiol and Thio-ether (2474.2 eV), G3: Sulfinic acid and Sulfoxide S (2476.3 eV), G4: Intermediate S forms, Sulfonate and Sulfonic acid (2481.0 eV), and G5: Oxidised S forms, Sulfate S and Sulfate ester (2482.7 eV)

Series	Treatment	Year	G1 [RSSR]	G2 [RSH & RSR]	G3 [RSO(OH) & RS(=O)R]	G4 [$RS(=O)_2O^-$ & $RS(=O)_2OH$]	G5 [$ROSO_3$ & SO_4^{2-}]	C-bonded S[1]
Broadbalk wheat	*Control*	1865	5.9	14	6.0	24	50	50
		1914	9.6	14	5.2	25	46	54
		1944	5.9	14	6.0	27	47	53
		1987	9.5	17	6.7	25	42	58
		2010	5.9	14	5.8	28	46	54
	FYM	1865	2.7	6.9	3.1	18	70	30
		1914	8.5	22	9.2	25	35	65
		1944	8.4	21	8.8	27	35	65
		1987	8.8	24	10	26	31	69
		2010	9.2	25	11	25	30	70
	NPKMgS	1865	4.1	8.5	3.8	24	59	41
		1914	10	14	5.3	25	46	54
		1944	5.3	13	5.5	28	49	51
		1987	9.2	14	5.0	27	45	55
		2010	5.7	13	5.7	28	47	53
Wilderness	*Broadbalk*	1881	6.2	13	5.5	25	50	50
		1904	9.1	15	5.6	25	45	55
		1964	7.1	16	7.4	27	42	58
		1999	7.3	22	9.5	27	34	66
	Geescroft	1883	13	18	6.5	24	39	61
		1904	10	16	6.0	26	41	59
		1965	5.2	13	5.2	24	52	48
		1999	7.6	25	9.3	23	35	65

1 – Sum of G1 to G4

Supplementary Table S4 Matrix of principal components computed by PCA for total SOC, total S, C:S ratio and the S composition determined from XANES spectra.

Variable	PC1 (60.3%)	PC2 (17.4%)
Total SOC	0.35 [x]	-0.44
Total S	0.31[x]	-0.45
C:S ratio	0.21 [x]	-0.10
G1 [RSSR]	0.20 [x]	0.54
G2 [RSH & RSR]	0.42 [x]	0.04
G3 [RSO(OH) & RS(=O)R]	0.42 [x]	-0.05
G4 [RS(=O)$_2$O$^-$ & RS(=O)$_2$OH]	0.12	0.46
G5 [SO_4^{2-} & $ROSO_3$]	-0.40 [x]	-0.27
C-bonded S: Oxidised S	0.42 [x]	0.10

[x] - $p < 0.05$

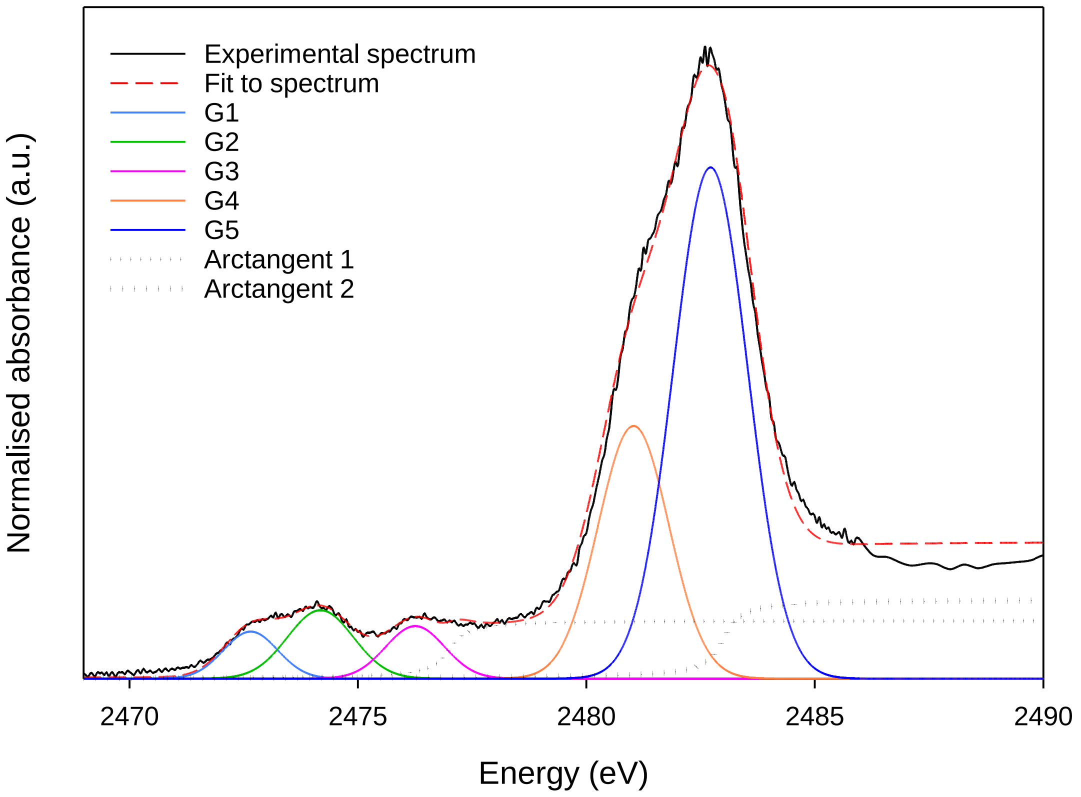

Supplementary Figure S1 Peak fitting of XANES spectra showing the five Gaussian peaks for each functional group (G1 2472.6 eV, G2 2474.2 eV, G3 2476.3 eV, G4 2481.0 eV, G5 2482.7 eV), and two arctangent functions (2477 and 2483 eV) representing the edge steps of reduced and oxidized S respectively.

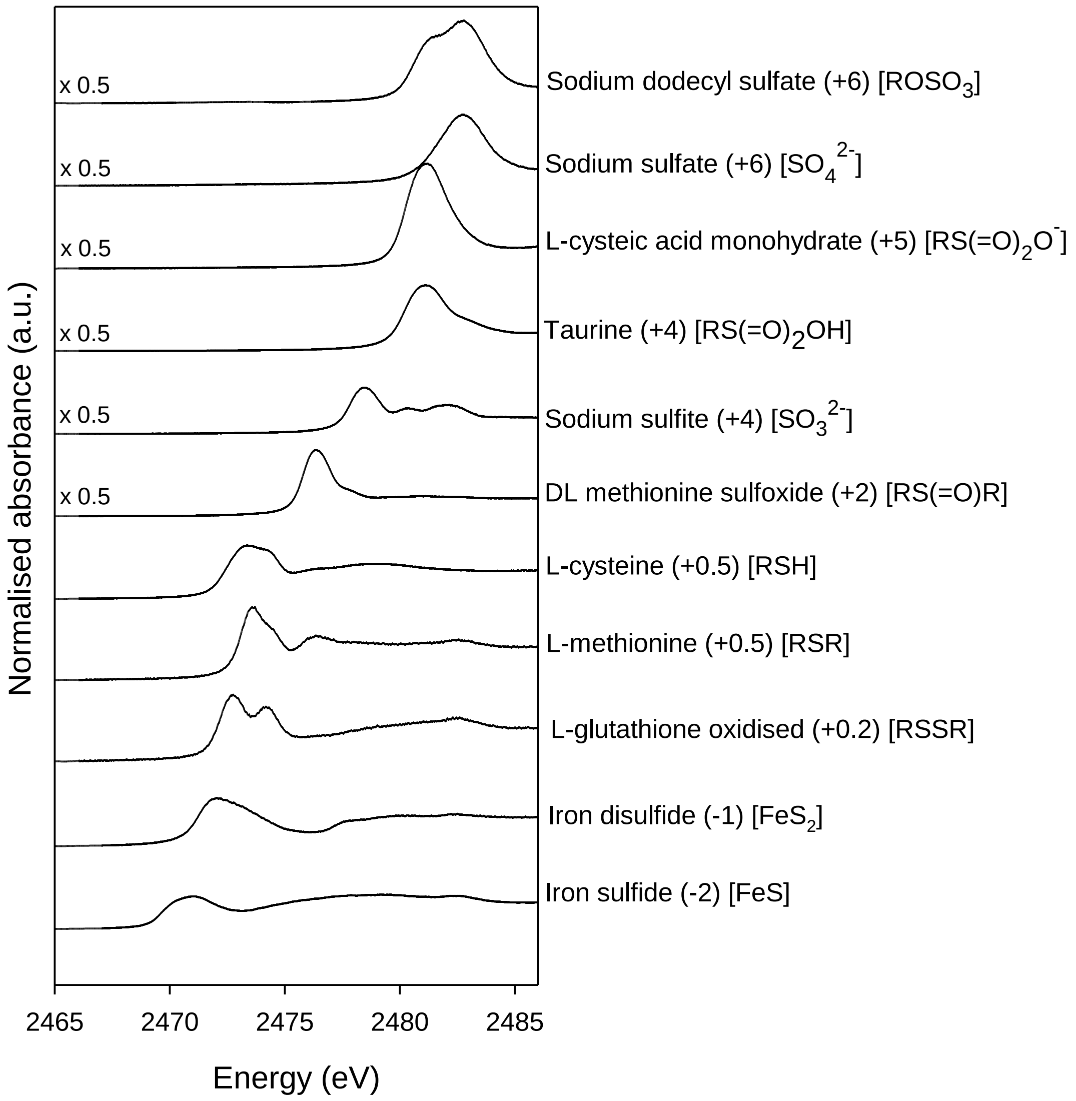

Supplementary Figure S2 Normalised S K-edge XANES spectra for selected organic and inorganic sulfur standards. Spectra are offset vertically to allow for comparison.

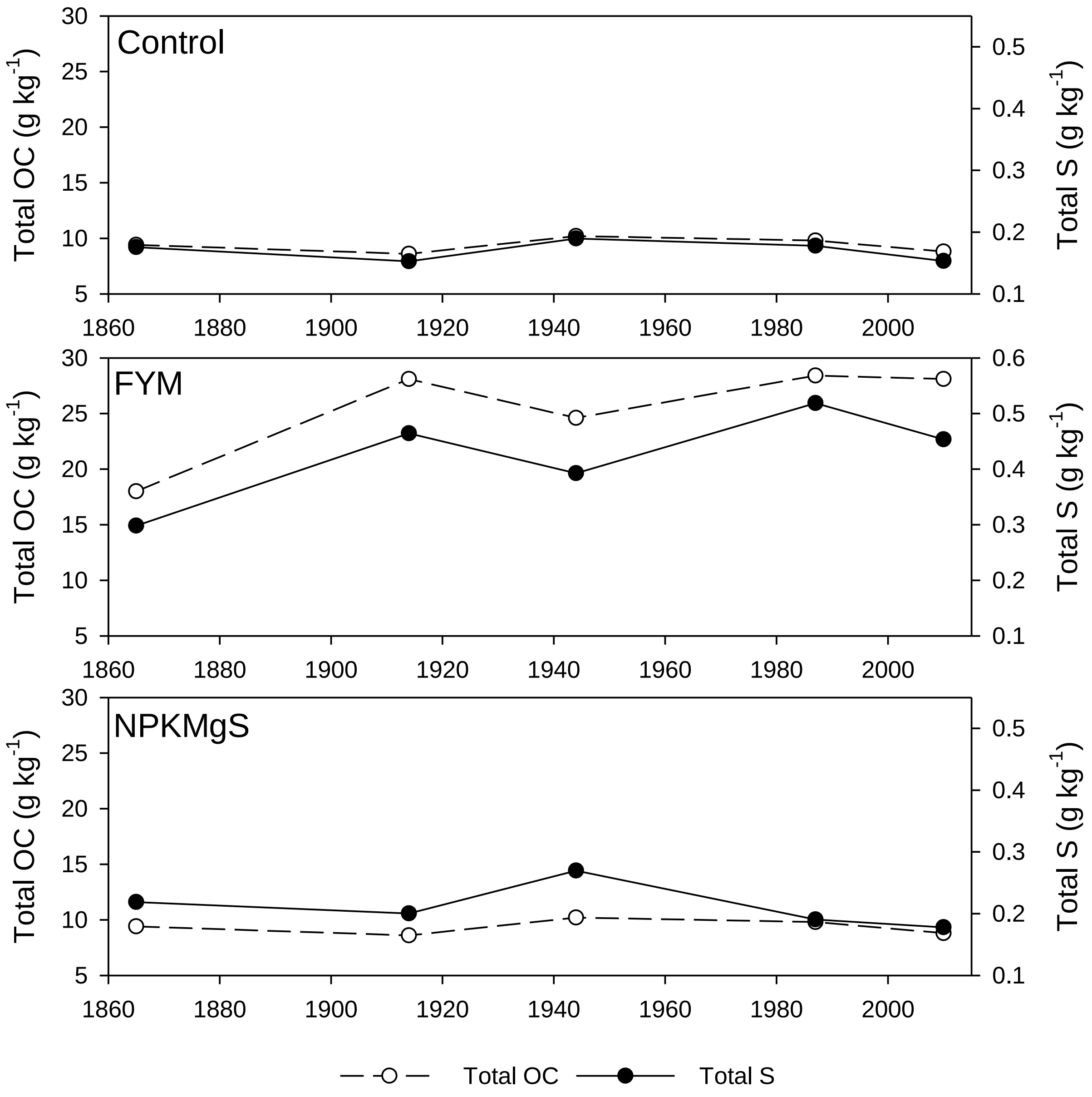

Supplementary Figure S3 Total OC and sulfur over time in the different treatments of the Broadbalk Wheat Experiment.

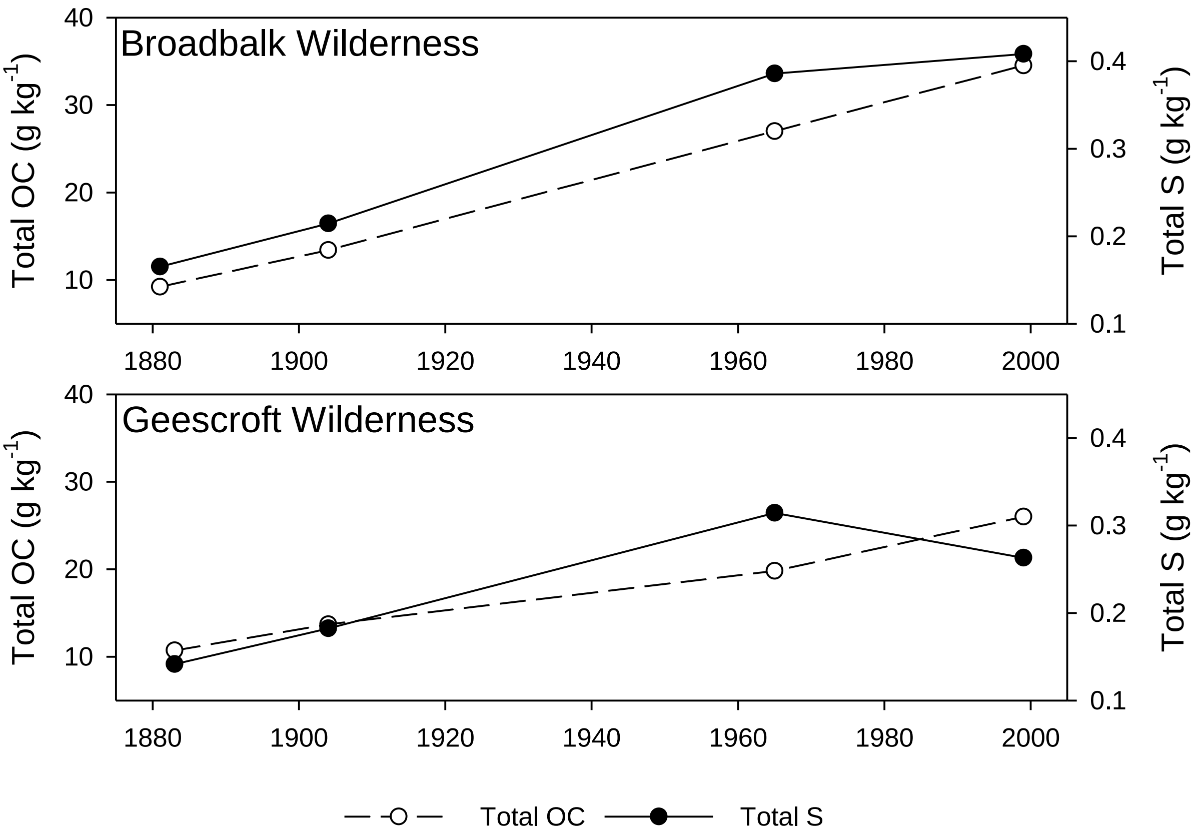

Supplementary Figure S4 Total OC and sulfur over time in the Broadbalk and Geescroft Wilderness sites

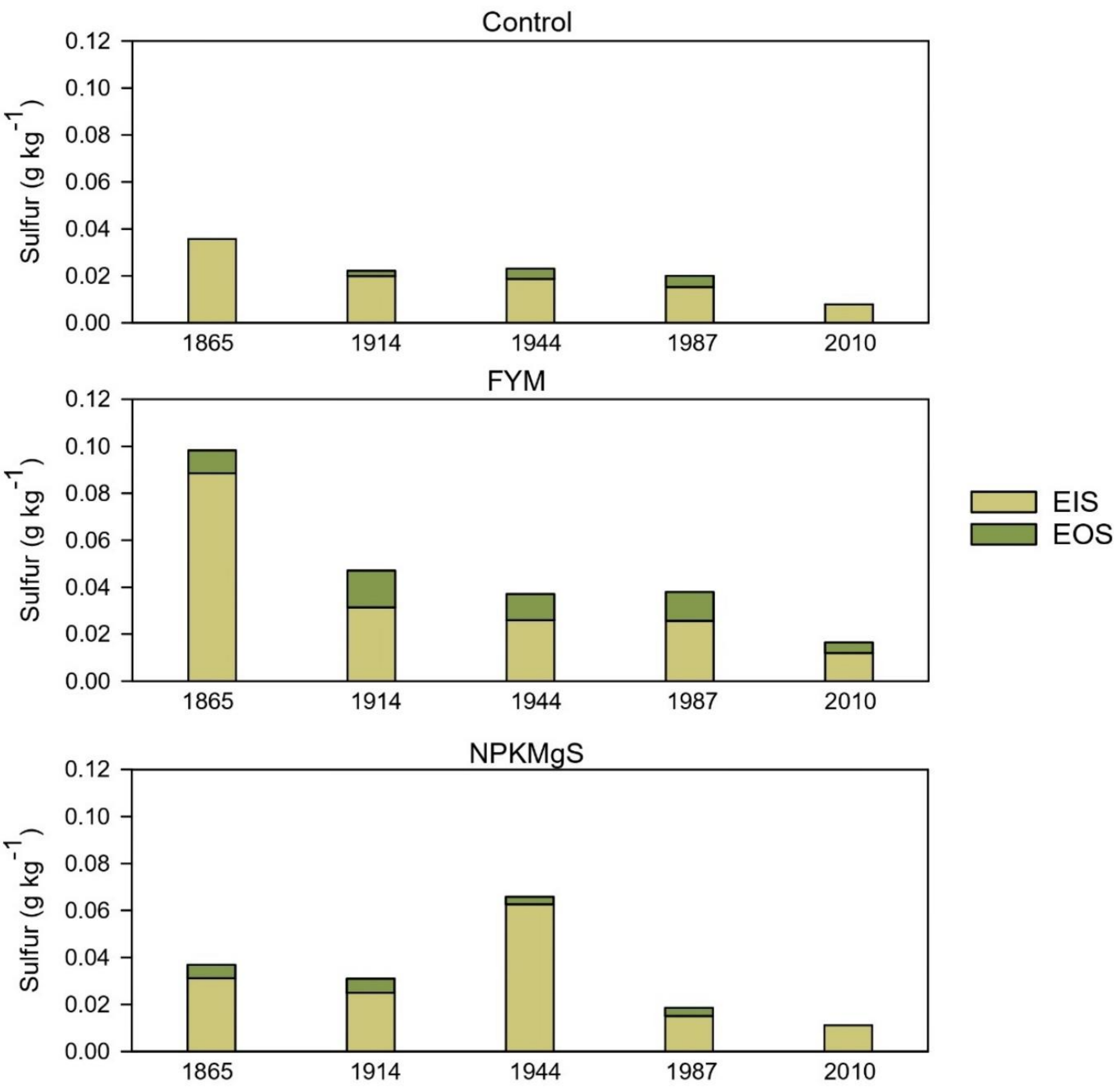

Supplementary Figure S5 Extractable inorganic sulfur (EIS) and extractable organic sulfur (EOS) over time in the different treatments of the Broadbalk Wheat Experiment.

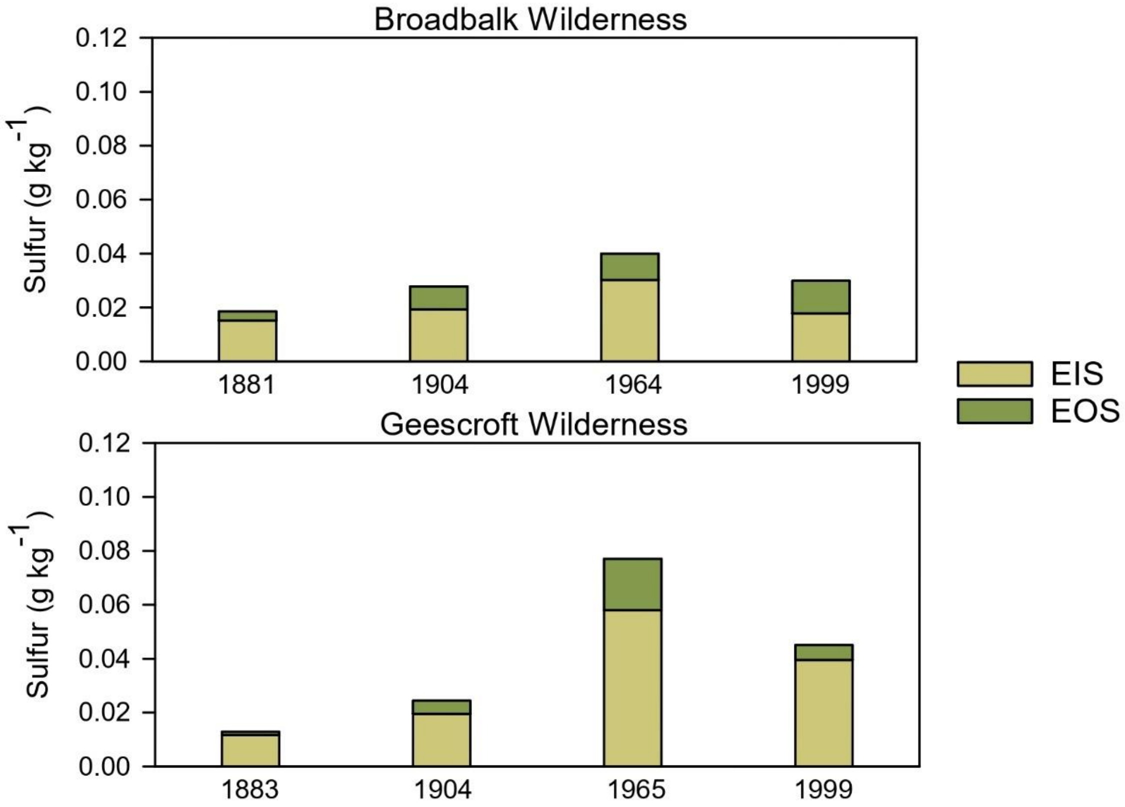

Supplementary Figure S6 Extractable inorganic sulfur (EIS) and extractable organic sulfur (EOS) over time in the Broadbalk and Geescroft Wilderness sites
